# Influence of the Periodontal Disease, the Most Prevalent Inflammatory Event, in Peroxisome Proliferator-Activated Receptors Linking Nutrition and Energy Metabolism

**DOI:** 10.3390/ijms18071438

**Published:** 2017-07-05

**Authors:** Lourdes Román-Malo, Pedro Bullon

**Affiliations:** Laboratorio de Investigacion, Departamento de Estomatologia, Universidad de Sevilla, c/ Avicena s/n, Sevilla 41009, Spain; lvrmalo@gmail.com

**Keywords:** periodontitis, inflammasome, mitochondria, autophagy, atherosclerosis, diabetes, metabolic syndrome

## Abstract

Periodontal disease is considered one of the main pathologic diseases occurring in humans. Its pathologic process involves inflammatory reactions producing periodontal bone resorption and the tooth loss. But some patients do not present an evident clinical inflammation with bone resorption, and in others, the inflammation is prominent without bone resorption. A key question could be to investigate a different way of responding to aggression. Inflammation requires a complex intracellular metabolic process, starting with the harmful recognition and activation of the inflammasome, continues the energy supply with the alteration of oxidative stress conditions, and finishes with the elimination of the aggression with autophagy/apoptosis mechanisms, then concludes with recovery. Peroxisome proliferator-activated receptors (PPARs) are essential molecules produced in inflammation, and its genes and its activation have been related to periodontal disease. Also, an important aspect is the influence of PPARs in bone metabolism; the main periodontitis symptom is bone loss and PPARγ activation that can downregulate the bone resorption in experimental periodontitis, PPARγ-coated titanium dental implant surfaces could carry the antiinflammatory gene and restrain inflammation. PPARs could be one of the meeting background points with atherosclerosis/cardiovascular disease, diabetes and metabolic syndrome showing a modified proinflammatory statement such as it is described in periodontitis.

## 1. Introduction

Plaque-induced periodontal diseases are mixed infections which cause inflammation in the tissues around the teeth; this site is known as the periodontium and compromises all of its components. We divided it into two general categories based on whether illness to periodontium is reversible or not. So, respectively, we can differentiate between gingivitis and periodontitis [1].

**Figure 1:**
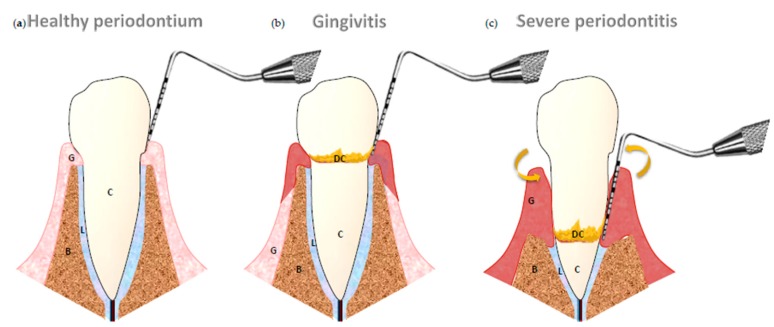
(**a**) Healthy periodontium: gingiva (G), periodontal ligament (L), root cementum (C) and alveolar bone (B); (**b**) Gingivitis; there is presence of calculus (DC). The inflammation is reversible; and (**c**) Severe form of chronic periodontitis: gingival inflammation, depth pocket, subgingival calculus and mobility.

Gingivitis supposes the presence of gingival inflammation without loss of gingival attachment; the site affected is limited to the gingiva. On the other hand, periodontitis is an inflammatory disease of the periodontal tissues with a more complex pathogenic process. Main symptoms in periodontitis are an apical migration of gingival attachment, a progressive periodontal bone loss and the formation of a periodontal pocket. The endpoint in the progression of this process is the tooth loss. This disease does not compromise the life of the patient, but produces a decrease in their quality of life. Severe periodontitis may result in discomfort for the patient, tooth mobility, abscess formation, occasional pain, difficulty in chewing and finally, tooth loss [2] (Figure 1).

Nowadays, periodontal diseases take real importance in our society on account of their high prevalence. The prevalence of gingivitis is over 80% with the peak being reached during puberty [3]. Chronic periodontitis affects about 50% of the European population, and over 10% may suffer from an aggressive form, with this prevalence increasing up to 70–85% at 60–65 years of age [4]. Also, it is expected that this prevalence data is maintaining during the next decade in Europe [5]. Therefore, periodontal diseases are considered one of the most common diseases.

The key questions are the etiological and pathogenic processes involved, but many aspects are still unclear. Authors try to explain why in some patients, the disease produced by bacterial infection is limited to the gingiva and in others ones produces bone loss, and also the degree of bone resorption could be more or less severe, and even in aggressive periodontitis a lack of plaque and inflammation is described.

To explain it, researches have been focused on individual factors like the host response or microbial infection. Traditionally, the main cause of periodontal diseases is attributed to some bacterial species that colonize subgingival sites or pockets, which are the responsible for the inflammatory reaction. Nowadays, our treatment consists of reducing and eliminating the subgingival microorganisms. But, we have to recognize some significant limitations: we do not obtain success in all the patients, prevention is not feasible, we do not have any ways to identify the susceptible population, and a complete regeneration of periodontal tissues is presently impracticable. Besides, plaque is not essential in aggressive forms of periodontitis, and the control of the periodontal disease becomes more difficult [6]. Certain microorganisms are related with the pathogenesis of periodontal disease, but the evidence does not support the invasion of a periodontal pathogen as a key step in this process [7]. Therefore, periodontitis is produced by an imbalance among microbiota/dental biofilm and the inflammatory response that have to play a key role.

Inflammation is the main common backgrounds, with others systemic diseases such as atherosclerosis and atherosclerosis, diabetes and metabolic syndrome. These have been correlated with periodontitis in epidemiological studies. But, while periodontitis does not kill people, these others pathologies jeopardize the life of patients and are the main causes of death in our countries. All have a complex etiology, with a combination of non-modifiable risk factors (age or genetic), and modifiable risk factors: tobacco, alcohol, malnutrition, and chronic infection [8].

Inflammation is a physiological response to stress which involves a multitude of processes. Firstly, it is necessary that innate immune system recognizes the aggression made by microorganisms, toxins and chemical compounds. These are identified as the pathogen-associated molecular patterns (PAMPs) or danger-associated molecular patterns (DAMPs) that are recognized via the pattern recognition receptors (PRRs) [9]. The cytoplasmic PAMPs and endogenous DAMPs activate PRRs and induce an intracellular signaling leading to the activation of inflammasome-driven inflammation [10]. Inflammasomes are large multiprotein complexes localized in the cytoplasm of the cell; it is essential in initiating innate immune responses. They produce the maturation of pro-inflammatory cytokines and the activation of inflammatory cell death [11]. Several inflammasomes are well-characterized for their role in recognition of PAMPs and DAMPs such as NLRP3 (nucleotide-binding leucine-rich repeat containing family, pyrin domain containing 3) [12]. Inflammasome activity is central to appropriate host defense and protection from sepsis, but excessive inflammasome activation could be detrimental to health [13].

Secondly, to maintain this inflammatory reaction, a sufficient supply of energy obtained from nutrients of the diet is necessary. But nowadays, humans have an excessive offer of nutrients that produce metabolic disorders. In the origin of these is a “proinflammatory” state produced by excessive caloric intake and over-nutrition and other innate conditions related with inflammation [14]. These nutrients are metabolized via the Krebs cycle, fatty-acid oxidation and amino acid oxidation to obtain adenosine 5´-triphosphate (ATP). This ATP is the fuel in cells of living organisms and the mitochondrion is the major source of ATP via cell respiration [15]. During respiration, oxygen is used to oxidize molecules rich in carbon and hydrogen, and a reduction of oxygen to water. This reaction realizes free radicals and/or reactive oxygen species (ROS); both are capable of damage different substrates. The antioxidants are responsible for preventing the oxidative damage, but any defect in the respiratory chain, mitochondrial diseases and age could alter this balance and produce oxidative stress [16]. Also, we cannot forget free radicals and ROS are essential in the physiological processes, but when an antioxidant system is unable to counteract their action efficiently, tissue and cell damage result [17]. So, the role of oxidative stress is essential in the mechanisms of inflammatory condition [14].

Finally, to maintain and control inflammation, cells need to function properly. The degradation of organelles and the elimination of invading pathogens are essential for the cell’s homeostasis. In this sense, one of the main catabolic pathways is autophagy. Autophagy allows for an appropriate response to stress and extrinsic or intrinsic insults [18]. Impaired autophagy produce various pathologies, including cardiovascular disease and periodontitis [19,20], and is directly related to a regulatory role for ROS originated in mitochondria [21]. When autophagy is blocked, this leads to activation the NLRP3 inflammasome, which explains the frequent association of mitochondrial damage with inflammatory diseases [22]. Additionally, autophagy is regulated by the mammalian target of rapamycin (mTOR), which shuts off autophagy in the presence of growth factors and abundant nutrients [23]. In vitro surveys suggest that periodontal pathogens can induce mTOR degradation in epithelial cells [24]. Furthermore, the mTOR activates peroxisome proliferator-activated receptors (PPARs) [25], which have been increasingly recognized as key players in the pathogeneses of metabolic diseases and primary metabolic regulators [26]. In addition, PPARs are in direct relation with the NLRP3 inflammasome [27].

It is well-known that periodontitis shares pathological features with the mentioned systemic pathologies [7]. The evidence appears to indicate that the common link between periodontitis and systemic diseases seems to be inflammation [9]. Although inflammation is a protective physiological response, it can sometimes lead to the destruction of tissue, starting a disease. There are a multitude of molecular routes related with inflammation which have been studied to explain the physiopathological processes taking place place in periodontitis. Routes related with inflammasomes and autophagy have been widely investigated [9,19]. Also, there is evidence for a decreased antioxidant capacity and mitochondrial dysfunction in patients with periodontitis [28,29]. In fact, animals studies have shown that antioxidant treatment with coenzyme Q is able to reduce the progression of alveolar bone destruction [30].

Nowadays, the key questions in periodontitis remain unanswered. Why in some patients does the inflammatory response cause a pathology, and why not in another? With the recent knowledge we have gained, we have began to look for new keys in this process and to try to obtain an answer within this new family of receptors known as PPARs.

## 2. Peroxisome Proliferator-Activated Receptors (PPARs) in Periodontitis as Inflammatory Disease

PPARs were originally identified in *Xenopus* frogs as receptors that induce the proliferation of peroxisomes in cells. They are a group of nuclear receptor proteins regulating the expression of genes. Three types of PPARs have been identified: α, γ, β/δ. PPARα is expressed in expressed in liver, kidney, heart, muscle, and adipose tissue. PPARγ is expressed in all tissues. PPARβ/δ is strongly expressed in brain, adipose tissue and skin. They are key players in the regulation of cellular differentiation, development, and metabolism (carbohydrate, lipid, protein), and tumor production [31]. Basically, PPARs are considered as lipid sensors, that according to its availability leads to fatty acid metabolism or lipid storage. Also, PPARs play a major role in inflammation. For instance, they are involved in age-related inflammation, caloric restriction, and longevity [32]. Activation of PPARγ reduces the inflammation and can be activated by some non-steroidal anti-inflammatory and oral antidiabetic drugs. Many of PPARγ’s established anti-inflammatory effects have been shown to occur through innate immune signaling, particularly in monocytes and macrophages, but new data is investigating the role of PPARγ and its ligands (including thiazolidinediones, prostaglandins, and oleanolic acids) in the resolution of inflammation [33]. Genetically modified mice showed that PPARα-impaired activity results in a modified inflammatory reaction, which causes a temporary delay in epidermal healing [34]. The upregulation of PPARβ/δ expression and endogenous agonist is triggered by tumor necrosis factor-α (TNF-α), a proinflammatory cytokine [35]. The antiinflammatory role of PPARβ/δ has been related to the utilization of agonists in different models of neurodegenerative diseases [36]. In inflammatory bowel diseases, the activation of PPARα or PPARγ has antiinflammatory effects in the intestine, decreasing the production of inflammatory markers and slowers the progression of colitis [37]. PPARα is the molecular target of the fibrates class of drugs, such as fenofibrate, which act as agonistic ligands of PPARα [38]. Also, PPARα ligands can inhibit the expression of various proinflammatory genes, such as interleukin (IL)-6, vascular cell adhesion molecule-1, platelet-activating factor (PAF) receptor and cyclooxygenase (COX)-2 generating prostaglandin E2 (PGE2) and thromboxane B2 (TxB2), in response to cytokine activation [39].

On the whole, PPARs ligands activate PPARs to express their target genes. These agonists can be synthetic molecules, such as anti-hypertriglyceridemia drugs and insulin resistance, or fatty acids and eicosanoids. The first synthetic high-affinity ligand for PPARβ/δ is the GW0742; it can modulate the inflammatory process and could limit the development of periodontitis. It has been demonstrated in rats to result in a substantial reduction of ligature-induced periodontitis, reducing plasma extravasation and the degree of bone resorption. Therefore, it could provide a promising approach for the treatment of periodontitis, reducing plasma extravasation and the bone resorption during periodontitis [40].

**Figure 2:**
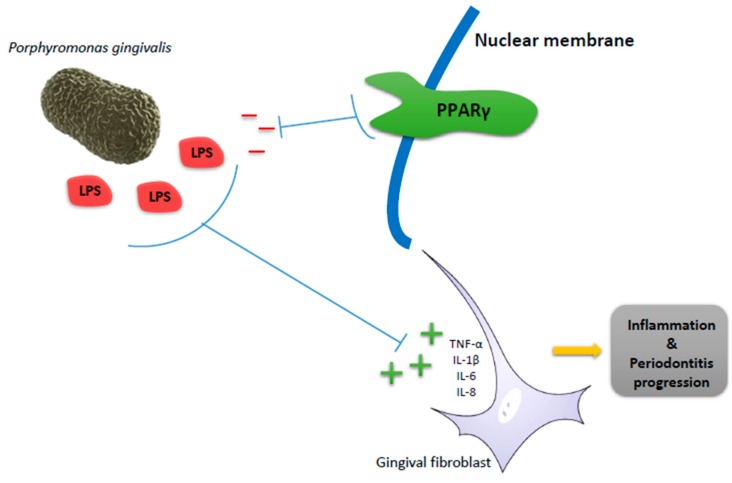
Anti-inflammatory effect of peroxisome proliferator-activated receptors γ (PPARγ). A rat model of ligature-induced periodontitis shows the effects of WY14643, a potent peroxisome proliferator activator receptor α (PPARα) agonist. The treatment with WY14643 attenuates the degree of inflammatory response in the gingival tissues. All of these data support that PPARα has a pernicious role in the development of damage associated with periodontitis in rats [50]. TNF-α, tumor necrosis factor-α; IL-1β, interleukin 1 β; IL-6, interleukin 6; IL-8, interleukin 8; and LPS, lipopolysaccharide.

At present, therapy with mesenchymal stem cells (MSC) is one of the most promising options to treat some diseases. Periodontal ligament stem cells have emerged as readily available therapeutic sources. They share core MSC properties such as colony formation and multipotential differentiation abilities, and may be ideal for repairing periodontal defects and achieving tooth regeneration with fewer drawbacks. Real-time PCR shows a highly elevated level of PPARγ in these cells [41]. One of the main bacteria involved in periodontitis is *Porphyromonas gingivalis*, and its lipopolysaccharide (LPS) induce the expression of inflammatory cytokines TNF-α, IL-1β, and IL-6 and chemokines IL-8 in human gingival fibroblasts (HGF) [42]. The activation of PPARγ could inhibit LPS-induced inflammatory response [43] (Figure 2). A major metabolite of anthocyanins, the protocatechnic acid, inhibit LPS-induced inflammation, suppressed IL-6 and IL-8 production in LPS-stimulated HGF and can be reversed by the PPARγ antagonist GW9662 [44]. The evidence that rosiglitazone (RGZ), a PPARγ agonist, reduces acute and chronic inflammation has been tested in a rat model of periodontitis. It demonstrated a reduction in the development of periodontitis, the presence in the mucogingival tissues with polymorphonuclear cells, the degree of nitrotyrosine formation and the level of injury in mucogingival tissues. All these findings support an attenuation of the damage produced in experimental periodontitis [45]. The interaction between PPARγ gene polymorphism with periodontal disease has been studied in some papers without a clear conclusion. The association of five polymorphisms (rs10865710, rs2067819, rs3892175, rs1801282, rs3856806) within the PPARG gene with chronic periodontitis was studied in a group of patients aged from eight to 85 years-old. The results show that none of the haplotypes was significantly different between the periodontitis and healthy controls. Therefore, it could not demonstrate a significant association between PPARG gene variants and chronic periodontitis [46]. In a group of pregnant women (the average age of the subjects being approximately 31 years-old), it could not be demonstrated an association between the PPARγ Pro12Ala polymorphism and the presence of periodontitis because they did not reach statistical significance [47]. Later the same group of authors showed a significant association with periodontal disease in obese elderly females, indicating a gene–gene or gene–environmental interaction among periodontal disease and each gene polymorphism [48]. Another study made in a group of postmenopausal women with slight periodontitis do not demonstrate any independent associations between the PPARγ Pro12Ala polymorphism and any of the periodontal clinical parameters [49].

## 3. PPARs in Periodontitis as Bone Disease

The balance of bone metabolism is supported by osteoclast-induced bone resorption and osteoblast-induced bone formation. Osteoblastic lineage cells and activated T lymphocytes produce a receptor activator of nuclear factor (NF-κB) ligand (RANKL). It is the essential factor for osteoclast formation, fusion, activation, and survival, resulting in bone resorption and bone loss. RANKL has a specific receptor located on osteoclast and dendritic cells: RANK. The activation of RANK promotes the signaling cascade including stimulation of the c-jun, NF-κB, and protein serine/threonine kinase (PKB/Akt) pathways. The effects of RANKL are counteracted by osteoprotegerin (OPG) which acts as a soluble neutralizing receptor. The regulation of RANKL and OPG are mediated by cytokines (tumor necrosis factor α, interleukins 1, 4, 6, 11, and 17), various hormones (glucocorticoids, vitamin D, estrogen), and several mesenchymal transcription factors (such as Cbfa-1, peroxisome proliferator-activated receptor γ, and Indian hedgehog).

Stimulation of PPARγ induces the development of mesenchymal stem cells into adipocytes instead of osteoblasts and results in the decrease of the number of osteoblasts and bone mineral density (BMD). PPARγ gene polymorphisms have been reported to be associated with bone loss and osteoporosis [51]. A study showed that PPARγ also functions as an essential modulator of osteoclast differentiation. Mice with PPARγ-deficiency in their hematopoietic lineage cells are osteopetrotic as a result of impaired c-Fos expression. These data suggest that PPARγ acts as an important regulator of c-os expression [52]. Another study demonstrates that the activation of liver X receptor (LXR) attenuated the PPARγ agonist-induced c-Fos expression, and the ectopic expression of LXR suppressed PPARγ-induced Activator protein 1 (AP-1) promoter activity. LXR can be identified as a negative regulator of PPAR-mediated induction of c-Fos and transactivation of AP-1 in the context of osteoclast development. The authors propose that LXR agonists may be of value in the treatment of bone-erosive diseases characterized by increased osteoclast number [53].

Mechanical loading is essential in the maintenance of bone mass and structures around the teeth and bone physiology. Some papers indicate that the mechanical loading of osteoblasts produces the expression of transcription factor PPARγ-1 mRNA. The coordinated synthesis of Δ^12^PGJ_2_, a natural ligand for PPARγ-1, with the increased expression of PPARγ-1, point out that the biomechanical transduction pathways that initially involve the activation of cyclooxygenases may also require the activation of the Δ^12^PGJ_2_–PPAR pathway [54]. These data also suggest that prostaglandin (PGD2) biosynthesis induced in bone cells by mechanical stress may be one of the major inducers of osteogenesis. The same group of authors investigated the molecular events produced in osteogenesis secondary to mechanical loading and Δ^12^PGJ_2_, namely the induction of bone morphogenetic proteins and peroxisome proliferator-activated receptor γ-1, a nuclear receptor for Δ^12^PGJ_2_. Mechanical strain increased the mRNA expression of peroxisome proliferator-activated receptor γ-1. In stretched cells, bone peroxisome proliferator-activated receptor γ-1 expression was blocked by cyclooxygenase inhibitors, but improved by exogenous Δ^12^PGJ_2._ These data suggest that the Δ^12^PGJ_2_/peroxisome proliferator-activated receptor γ-1 is essential in osteogenesis [55]. The effect of Δ^12^PGJ_2_ on periodontitis inhibited the production of interleukin-6 (IL-6) in osteoblast-like cells MC3T3E-1 incubated with LPS. Data showed that it is an effective inhibitor of the LPS-stimulated IL-6 production, in a dose-dependent manner, through the blockade of NF-κB and the Akt pathway. These data suggest a possible mechanism responsible for its anti-inflammatory action and the use as a therapeutic drug for treating LPS-stimulated periodontal diseases [56].

A relationship between osteoporosis and periodontal conditions has been studied. Systemic bone density and oral infection, analyzed in the presence of a periodontal bacteria such as *Tannerella forsythensis*, was found to modify oral bone loss in post-menopausal women aged <70 years [57]. In a group of 400 postmenopausal women, osteoporosis was significantly associated with severe alveolar crestal bone loss and the prevalence of periodontitis cases [58]. Another paper showed data from 90 women aged 45–70 years; the osteoporotic women presented severe periodontitis with greater gingival inflammation, greater clinical attachment level and greater gingival recession than the women with normal BMD [59]. A group of 179 male and female patients were divided into an osteopenia, and a non-osteopenia group and a follow-up clinical surveys were done by measuring periodontal conditions after 3 years. BMD was related to the number of progressive sites which had additional attachment loss during the observational period [60].

Pathogenic bone resorption occurring in periodontal disease is known to be produced by inflammation, PPARγ activation can downregulate the bone resorption mediated by RANKL-dependent osteoclastogenesis. RGZ, a high-affinity synthetic agonist for PPARγ, influences the bone resorption induced in rats by experimental periodontitis caused by ligature placement around the tooth. Histological and immunohistochemical analysis showed that the treatment with RGZ decreased bone resorption, alongside reduced RANKL expression, in contrast to those animals treated with empty vehicles. Also, along with these results obtained from in vivo experiments, RGZ also suppressed in vitro osteoclast differentiation in the presence of RANKL in mouse monocyte/macrophage (MOCP-5) osteoclast precursor cells, and the downregulation of the expression of RANKL-induced TRAP mRNA. So, RGZ might suppress the bone resorption by inhibiting RANKL-mediated osteoclastogenesis obtained during experimental periodontitis in rats [61].

A study investigated the potential associations between the PPARγ Pro12Ala polymorphism, periodontitis and bone mineral density (BMD) in 674 women between the ages of 55 and 74 years-old. The association between BMD or periodontitis and PPARG Pro12Ala polymorphism was not independent. The common point could be in the modulator role of the polymorphism in this relationship [62].

In dentistry, the treatment with implants involved the bone integration of the titanium surface without any inflammation. Some papers have been proposed to deliver antiinflammatory molecules PPARγ on titanium surfaces using chitosan gold nanoparticles (Ch-GNPs). They evaluated the protein levels of TNF-α and IL-1β in RAW 264.7 cells. Expression of these cytokines was not observed in cells cultured on Ch-GNPs/PPARγ-coated titanium surfaces. But, upregulation of pro-inflammatory cytokines was detected on non-coated and Ch-GNPs/LacZ-coated titanium surfaces. These data suggested that the Ch-GNPs/PPARγ coated titanium surfaces could carry the anti-inflammatory gene and inhibit inflammation [63].

## 4. PPARs in Periodontitis and Its Related Systemic Diseases

We have shown that periodontitis is related to some systemic diseases such as cardiovascular disease produced by atherosclerosis, diabetes mellitus, and metabolic syndrome. The pathophysiology of these diseases share a common background of low-grade modified inflammation. Periodontitis results from an imbalance of the bacteria and the host inflammatory response that produce an irreversible alveolar bone resorption, leading to tooth loss. Nowadays, the key question is why in some patients, the inflammation is limited to gingivitis as a reversible disease, and in others produces periodontitis with an irreversible alveolar bone loss. One of the possible mechanisms is an impaired inflammatory response. We have proposed that the interrelationship could lie at a cellular level, namely oxidative stress and mitochondrial dysfunction, as a meeting background among such chronic diseases and periodontitis [64]. Also, all the periodontitis-related systemic diseases show in its pathogenic mechanism an altered inflammation associated with an excessive caloric intake and increased or inadequate free fatty acid intakes [65]. Even the origins of susceptibility for these diseases in the adult can be identified to the early life, early-life nutrition plays an essential role in placental and fetal growth, organogenesis, and development [66]. PPARs contribute to the plasticity of the placenta and are under epigenetic control; it has been proposed there is a relationship between environmental factors, such as maternal nutrition, and the epigenetic regulation of PPRs [67]. Glucose and related sugars, lipids, and amino acids are essential nutrients. The control of the above environmental nutrient levels is essential for life; it is controlled by nutrient-sensing mechanisms and pathways. One of them is PPARs; they are nutrient-sensing nuclear receptors with integrators activity in metabolic responses [68].

Metabolic syndrome is defined as the combination of multiple metabolic disorders, including obesity, dyslipidemia, glucose intolerance, inflammation, and hypertension. Low-grade inflammation is a common background between insulin resistance, obesity and type-2 diabetes [65]. In recent years, some studies showed that PPARs could improve several of the metabolic abnormalities associated with the metabolic syndrome [69].

Specific genetic polymorphisms have been associated with a susceptibility to obesity. Expression of these susceptibility genes can be modified by epigenetic and environmental factors. Diet and nutritional status can affect the transcription factors and microRNAs (miRNAs) regulatory systems. Evidence from in vitro studies and from human studies has identified specific miRNAs associated with obesity. miRNAs are small, non-coding RNA molecules that bind and form complexes with mRNA species, namely to the 3’UTR of the mRNA [70]. miRNAs regulate adipocyte development and lipid metabolism. Expression of miR-27 suppresses the translation of peroxisome proliferator-activated receptor-γ (PPARγ) and is an essential early regulator of adipogenesis [71]. miR-143 expression is upregulated 3.3-fold in obese mice, and increases the adipocyte differentiation markers PPARγ and the adipocyte fatty-acid-binding protein [72]. PPARγ also regulates adipogenesis, and its polymorphisms have been suggested to be a risk for obesity. Body mass index (BMI) has been associated with Pro12Ala polymorphism [73]. Also, it was studied whether the Pro12Ala polymorphism of peroxisome proliferator-activated receptor γ (PPARγ) is related to insulin resistance, obesity, and weight loss and the potential interactions between fat intake and PPARγ polymorphism was explored. Data showed a protective role for the Ala12 allele against insulin resistance, and the interaction between dietary monounsaturated fatty acids (MUFA) and PPARγ2 for BMI [74]. PPARγ increased the plasma adiponectin concentration and is suggested as a therapeutic tool through the dietary regulation by derivatives of its active compounds, such as vitamins, polyunsaturated fatty acids (PUFA) or carotenoids [75].

The relationship between cardiovascular disease and PPAR has been reported. PPARβ/δ ligands and the overexpression of this nuclear receptor inhibits myocardial inflammatory responses, such as the lipopolysaccharide-mediated production of TNF-α. This had positive effects on animals that had undergone an ischemia/reperfusion injury or cardiac hypertrophy [76]. PPARβ/δ ligands are capable of improving cardiac function, and can even enhance some cardiac diseases such as heart failure, oxidative damage, hypertrophy, ischemia–reperfusion injury, lipotoxic cardiac dysfunction and lipid-induced cardiac inflammation [77].

The pathophysiology of diabetes includes an important inflammatory aspect. PPARα is essential in the dysregulation of inflammation. PPARα protein level was examined by immunohistochemistry in type-2 diabetic Goto-Kakizaki rats with induced periodontitis. The results showed the number of gingival fibroblast with PPAR-α protein was low at baseline and then in both, normoglycemic and diabetic rats with ligature-induced periodontitis. When the ligature was removed and the periodontitis solved, the normoglycemic rats presented a threefold increase in the relative expression of PPARα. On the contrary, no change was observed in the diabetic animals. However, diabetic rats treated with TNF-inhibitor, showed a significant increase, so that the diabetic rats with TNF-inhibitor function similarly to the normal animals. Thus, PPARα could participate in the resolution of periodontitis through its antiinflammatory properties, but under diabetic conditions, its upregulation is restrained [78].

The role of monocytes and macrophages in diabetic periodontal diseases has been investigated; the results indicated that monocytes and macrophages are important players in periodontal tissue inflammation and destruction in diabetic patients. Monocytes isolated from diabetic patients produce a greater amount of TNF-α and interleukin-1β in vitro than do nondiabetic controls [79]. A high glucose content markedly increased lipopolysaccharide-induced inflammatory cytokine and inducible nitric oxide synthase (iNOS) expression in U937 macrophages. These data indicated that hyperglycemia in diabetic patients may boost the inflammatory response leading to increased cytokine release. An antidyslipidemic drug, fenofibrate, increased lipopolysaccharide, induced TNF-α secretion and an antidiabetic drug, pioglitazone; increased lipopolysaccharide-induced TNF-α, interleukin-1β, and interleukin-6 secretion. But, pioglitazone and fenofibrate do not inhibit the stimulatory effect of high glucose and lipopolysaccharide on the secretion of pro-inflammatory cytokines effectively, but only if it suppressed by statin [80].

## 5. Conclusions

In the complex process involving inflammatory diseases such as periodontal disease, cardiovascular disease, diabetes and metabolic syndrome, PPARs could play an important role and give new clues to a better therapeutic approach. PPARs are the molecular targets of a number of marketed drugs. We should use those to prevent or treat periodontal diseases. This is our new challenge.

## Abbreviations

PAMPs	Pathogen-Associated Molecular Patterns
DAMPs	Danger-Associated Molecular Patterns
PRRs	Pattern Recognition Receptors
NLRP3	Nod Like Receptors Family, Pyrin Domain Containing 3
ATP	Adenosine 5´-Triphosphate
ROS	Reactive Oxygen Species
mTOR	Mammalian Target of Rapamycin
IL	Interleukin
COX	Cyclooxygenase
MSC	Mesenchymal Stem Cells
HGF	Human Gingival Fibroblasts
RGZ	Rosiglitazone
RANKL	Receptor Activator for Nuclear Factor κ B Ligand
NF-κB	nuclear factor kappa-light-chain-enhancer of activated B cells
OPG	Osteoprotegerin
BMD	Bone Mineral Density
Ch-GNPs	Chitosan Gold Nanoparticles
miRNAs	MicroRNAs
BMI	Body Mass Index
MUFA	Monounsaturated Fatty Acids
PUFA	Polyunsaturated Fatty Acids
TNF	Tumor Necrosis Factor

## References

[1] Armitage G.C. (2003). Diagnosis of periodontal diseases. J. Periodontol..

[2] Buset S.L., Walter C., Friedmann A., Weiger R., Borgnakke W.S., Zitzmann N.U. (2016). Are periodontal diseases really silent? A systematic review of their effect on quality of life. J. Clin. Periodontol..

[3] Jenkins W.M.M., Papapanou P.N. (2001). Epidemiology of periodontal disease in children and adolescents. Periodontol. 2000.

[4] König J., Holtfreter B., Kocher T. (2010). Periodontal health in Europe: Future trends based on treatment needs and the provision of periodontal services—Position paper 1. Eur. J. Dent. Educ..

[5] Madianos P., Papaioannou W., Herrera D., Sanz M., Baeumer A., Bogren A., Bouchard P., Chomyszyn-Gajewska M., Demirel K., Gaspersic R. (2016). EFP Delphi study on the trends in periodontology and periodontics in Europe for the year 2025. J. Clin. Periodontol..

[6] Armitage G.C. (2004). Periodontal diagnoses and classification of periodontal diseases. Periodontol. 2000.

[7] Mendes L., Azevedo N.F., Felino A., Pinto M.G. (2015). Relationship between invasion of the periodontium by periodontal pathogens and periodontal disease: A systematic review. Virulence.

[8] Ferretti F. (2015). Unhealthy Behaviours: An International Comparison. PLoS ONE.

[9] Santoni G., Cardinali C., Morelli M.B., Santoni M., Nabissi M., Amantini C. (2015). Danger- and pathogen-associated molecular patterns recognition by pattern-recognition receptors and ion channels of the transient receptor potential family triggers the inflammasome activation in immune cells and sensory neurons. J. Neuroinflamm..

[10] Schroder K., Tschopp J. (2010). The inflammasomes. Cell.

[11] Pedra J.H.F., Cassel S.L., Sutterwala F.S. (2009). Sensing pathogens and danger signals by the inflammasome. Curr. Opin. Immunol..

[12] Olsen I., Yilmaz Ö. (2016). Modulation of inflammasome activity by *Porphyromonas gingivalis* in periodontitis and associated systemic diseases. J. Oral Microbiol..

[13] Dagenais M., Skeldon A., Saleh M. (2012). The inflammasome: In memory of Dr. Jurg Tschopp. Cell Death Differ..

[14] Varela-López A., Quiles J.L., Cordero M., Giampieri F., Bullón P. (2015). Oxidative stress and dietary fat type in relation to periodontal disease. Antioxidants.

[15] Sies H. (1986). Biochemistry of oxidative stress. Angew. Chem. Int. Ed. Engl..

[16] Valko M., Leibfritz D., Moncol J., Cronin M.T.D., Mazur M., Telser J. (2007). Free radicals and antioxidants in normal physiological functions and human disease. Int. J. Biochem. Cell Biol..

[17] Battino M., Bullon P., Wilson M., Newman H. (1999). Oxidative Injury and inflammatory periodontal diseases : The challenge of anti-oxidants to free radicals and reactive oxygen species. Crit. Rev. Oral Biol. Med..

[18] Kroemer G., Mariño G., Levine B. (2010). Autophagy and the integrated stress response. Mol. Cell.

[19] Levine B., Kroemer G. (2008). Autophagy in the pathogenesis of disease. Cell.

[20] Bullon P., Cordero M.D., Quiles J.L., Ramirez-Tortosa M.D.C., Gonzalez-Alonso A., Alfonsi S., García-Marín R., de Miguel M., Battino M. (2012). Autophagy in periodontitis patients and gingival fibroblasts: Unraveling the link between chronic diseases and inflammation. BMC Med..

[21] Scherz-Shouval R., Elazar Z. (2007). ROS, mitochondria and the regulation of autophagy. Trends Cell Biol..

[22] Zhou R., Yazdi A.S., Menu P., Tschopp J. (2011). A role for mitochondria in NLRP3 inflammasome activation. Nature.

[23] Jung C.H., Ro S.-H., Cao J., Otto N.M., Kim D.-H. (2010). mTOR regulation of autophagy. FEBS Lett..

[24] Stafford P., Higham J., Pinnock A., Murdoch C., Douglas C.W.I., Stafford G.P., Lambert D.W. (2013). Gingipain-dependent degradation of mammalian target of rapamycin pathway proteins by the periodontal pathogen *Porphyromonas gingivalis* during invasion. Mol. Oral Microbiol..

[25] Blanchard P.-G., Festuccia W.T., Houde V.P., St-Pierre P., Brûlé S., Turcotte V., Côté M., Bellmann K., Marette A., Deshaies Y. (2012). Major involvement of mTOR in the PPARγ-induced stimulation of adipose tissue lipid uptake and fat accretion. J. Lipid Res..

[26] Tain Y.-L., Hsu C.-N., Chan J. (2015). PPARs link early life nutritional insults to later programmed hypertension and metabolic syndrome. Int. J. Mol. Sci..

[27] Lee H.J., Yeon J.E., Ko E.J., Yoon E.L., Suh S.J., Kang K., Kim H.R., Kang S.H., Yoo Y.J., Je J. (2015). Peroxisome proliferator-activated receptor-delta agonist ameliorated inflammasome activation in nonalcoholic fatty liver disease. World J. Gastroenterol..

[28] Baltacioğlu E., Akalin F. A., Alver A., Değer O., Karabulut E. (2008). Protein carbonyl levels in serum and gingival crevicular fluid in patients with chronic periodontitis. Arch. Oral Biol..

[29] Bullón P., Román-Malo L., Marín-Aguilar F., Alvarez-Suarez J.M., Giampieri F., Battino M., Cordero M.D. (2015). Lipophilic antioxidants prevent lipopolysaccharide-induced mitochondrial dysfunction through mitochondrial biogenesis improvement. Pharmacol. Res..

[30] Varela-Lopez A., Bullon P., Battino M., Ramirez-Tortosa M.C., Ochoa J.J., Cordero M.D., Ramirez-Tortosa C.L., Rubini C., Zizzi A., Quiles J.L. (2016). Coenzyme Q Protects against age-related alveolar bone loss associated to n-6 polyunsaturated fatty acid rich-diets by modulating mitochondrial mechanisms. J. Gerontol. Ser. A Biol. Sci. Med. Sci..

[31] Michalik L., Auwerx J., Berger J.P., Chatterjee V.K., Glass C.K., Gonzalez F.J., Grimaldi P.A., Kadowaki T., Lazar M.A., Rahilly S.O. (2006). International Union of Pharmacology. LXI. Peroxisome proliferator-activated receptors. Pharmacol. Rev..

[32] Chung J.H., Seo A.Y., Chung S.W., Kim M.K., Leeuwenburgh C., Yu B.P., Chung H.Y. (2008). Molecular mechanism of PPAR in the regulation of age-related inflammation. Ageing Res. Rev..

[33] Croasdell A., Duffney P.F., Kim N., Lacy S.H., Sime P.J., Phipps R.P. (2015). PPARγ and the innate immune system mediate the resolution of inflammation. PPAR Res..

[34] Michalik L., Feige J.N., Gelman L., Pedrazzini T., Keller H., Desvergne B., Wahli W. (2005). Selective expression of a dominant-negative form of peroxisome proliferator-activated receptor in keratinocytes leads to impaired epidermal healing. Mol. Endocrinol..

[35] Tan N.S. (2001). Critical roles of PPARβ/δ in keratinocyte response to inflammation. Genes Dev..

[36] Iglesias J., Morales L., Barreto G.E. (2017). Metabolic and inflammatory adaptation of reactive astrocytes: Role of PPARs. Mol. Neurobiol..

[37] Desreumaux P., Dubuquoy L., Nutten S., Peuchmaur M., Englaro W., Schoonjans K., Derijard B., Desvergne B., Wahli W., Chambon P. (2001). Attenuation of colon inflammation through activators of the retinoid X receptor (RXR)/peroxisome Proliferator-activated receptor γ (PPARγ) heterodimer. A basis for new therapeutic strategies. J. Exp. Med..

[38] Evans R.M. (1988). The steroid and thyroid hormone receptor superfamily. Science.

[39] Delerive P., de Bosscher K., Besnard S., Vanden Berghe W., Peters J.M., Gonzalez F.J., Fruchart J.C., Tedgui A., Haegeman G., Staels B. (1999). Peroxisome proliferator-activated receptor α negatively regulates the vascular inflammatory gene response by negative cross-talk with transcription factors NF-κB and AP-1. J. Biol. Chem..

[40] Di Paola R., Briguglio F., Paterniti I., Mazzon E., Oteri G., Militi D., Cordasco G., Cuzzocrea S. (2011). Emerging role of PPARβ/δ in inflammatory process associated to experimental periodontitis. Mediat. Inflamm..

[41] Kim K., Yi T., Yun J.-H. (2016). Maintained stemness of human periodontal ligament stem cells isolated following prolonged storage of extracted teeth. J. Periodontol..

[42] Andrukhov O., Ertlschweiger S., Moritz A., Bantleon H.-P., Rausch-Fan X. (2014). Different effects of *P. gingivalis* LPS and *E. coli* LPS on the expression of interleukin-6 in human gingival fibroblasts. Acta Odontol. Scand..

[43] Lu Y., Zhou Q., Shi Y., Liu J., Zhong F., Hao X., Li C., Chen N., Wang W. (2013). SUMOylation of PPARγ by rosiglitazone prevents LPS-induced NCoR degradation mediating down regulation of chemokines expression in renal proximal tubular cells. PLoS ONE.

[44] Wang Y., Zhou J., Fu S., Wang C., Zhou B. (2015). Preventive effects of protocatechuic acid on LPS-induced inflammatory response in human gingival fibroblasts via activating PPARγ. Inflammation.

[45] Di Paola R., Mazzon E., Maiere D., Zito D., Britti D., de Majo M., Genovese T., Cuzzocrea S. (2006). Rosiglitazone reduces the evolution of experimental periodontitis in the rat. J. Dent. Res..

[46] Folwaczny M., Manolis V., Markus C., Glas J. (2011). Variants of the human PPARG locus and the susceptibility to chronic periodontitis. Innate Immun..

[47] Hirano E., Sugita N., Kikuchi A., Shimada Y., Sasahara J., Iwanaga R., Tanaka K., Yoshie H. (2010). Peroxisome proliferator-activated receptor gamma polymorphism and periodontitis in pregnant Japanese women. J. Periodontol..

[48] Yoshihara A., Sugita N., Iwasaki M., Wang Y., Miyazaki H., Yoshie H., Nakamura K. (2015). The interaction between β-3 adrenergic receptor and peroxisome proliferator-activated receptor gamma gene polymorphism to periodontal disease in community-dwelling elderly japanese. J. Periodontol..

[49] Wang Y., Sugita N., Yoshihara A., Iwasaki M., Miyazaki H., Nakamura K., Yoshie H. (2014). PPARγ gene polymorphism, C-reactive protein level, BMI and periodontitis in post-menopausal Japanese women. Gerodontology.

[50] Briguglio E., di Paola R., Paterniti I., Mazzon E., Oteri G., Cordasco G., Cuzzocrea S. (2010). WY-14643, a potent peroxisome proliferator activator receptor-α PPARα agonist ameliorates the inflammatory process associated to experimental periodontitis. PPAR Res..

[51] Harsløf T., Tofteng C.L., Husted L.B., Nyegaard M., Børglum A., Carstens M., Stenkjær L., Brixen K., Eiken P., Jensen J.-E.B. (2011). Polymorphisms of the peroxisome proliferator-activated receptor γ (PPARγ) gene are associated with osteoporosis. Osteoporos. Int..

[52] Wan Y., Chong L.-W., Evans R.M. (2007). PPARγ regulates osteoclastogenesis in mice. Nat. Med..

[53] Kim H.-J., Yoon K.-A., Yoon H.-J., Hong J.M., Lee M.-J., Lee I.-K., Kim S.-Y. (2013). Liver X receptor activation inhibits osteoclastogenesis by suppressing NF-κB activity and c-Fos induction and prevents inflammatory bone loss in mice. J. Leukoc. Biol..

[54] Siddhivarn C., Banes A., Champagne C., Riche E.L., Weerapradist W., Offenbacher S. (2006). Prostaglandin D2 pathway and peroxisome proliferator-activated receptor γ-1 expression are induced by mechanical loading in an osteoblastic cell line. J. Periodontal Res..

[55] Siddhivarn C., Banes A., Champagne C., Riché E.L., Weerapradist W., Offenbacher S. (2007). Mechanical loading and Δ12prostaglandin J2 induce bone morphogenetic protein-2, peroxisome proliferator-activated receptor γ-1, and bone nodule formation in an osteoblastic cell line. J. Periodontal Res..

[56] Jung W.-K., Park I.-S., Park S.-J., Yea S.S., Choi Y.H., Oh S., Park S.-G., Choi I.-W. (2009). The 15-deoxy-Δ12,14-prostaglandin J2 inhibits LPS-stimulated AKT and NF-κB activation and suppresses interleukin-6 in osteoblast-like cells MC3T3E-1. Life Sci..

[57] Brennan-Calanan R.M., Genco R.J., Wilding G.E., Hovey K.M., Trevisan M., Wactawski-Wende J. (2008). Osteoporosis and oral infection: Independent risk factors for oral bone loss. J. Dent. Res..

[58] Al Habashneh R., Alchalabi H., Khader Y.S., Hazza’a A.M., Odat Z., Johnson G.K. (2010). Association between periodontal disease and osteoporosis in postmenopausal women in Jordan. J. Periodontol..

[59] Pepelassi E., Nicopoulou-Karayianni K., Archontopoulou A.D., Mitsea A., Kavadella A., Tsiklakis K., Vrotsos I., Devlin H., Horner K. (2012). The relationship between osteoporosis and periodontitis in women aged 45–70 years. Oral Dis..

[60] Yoshihara A., Seida Y., Hanada N., Miyazaki H. (2004). A longitudinal study of the relationship between periodontal disease and bone mineral density in community-dwelling older adults. J. Clin. Periodontol..

[61] Hassumi M.Y., Silva-Filho V.J., Campos-Júnior J.C., Vieira S.M., Cunha F.Q., Alves P.M., Alves J.B., Kawai T., Gonçalves R.B., Napimoga M.H. (2009). PPARγ agonist rosiglitazone prevents inflammatory periodontal bone loss by inhibiting osteoclastogenesis. Int. Immunopharmacol..

[62] Wang Y., Sugita N., Yoshihara A., Iwasaki M., Miyazaki H., Nakamura K., Yoshie H. (2013). Peroxisome proliferator-activated receptor (PPAR)γ polymorphism, vitamin d, bone mineral density and periodontitis in postmenopausal women. Oral Dis..

[63] Bhattarai G., Lee Y.H., Lee N.H., Park I.S., Lee M.H., Yi H.K. (2013). PPARγ delivered by Ch-GNPs onto titanium surfaces inhibits implant-induced inflammation and induces bone mineralization of MC-3T3E1 osteoblast-like cells. Clin. Oral Implant. Res..

[64] Bullon P., Newman H.N., Battino M. (2014). Obesity, diabetes mellitus, atherosclerosis and chronic periodontitis: A shared pathology via oxidative stress and mitochondrial dysfunction?. Periodontol. 2000.

[65] Dandona P., Aljada A., Bandyopadhyay A. (2004). Inflammation: The link between insulin resistance, obesity and diabetes. Trends Immunol..

[66] Rinaudo P., Wang E. (2012). Fetal programming and metabolic syndrome. Annu. Rev. Physiol..

[67] Lendvai A., Deutsch M.J., Plosch T., Ensenauer R. (2016). The peroxisome proliferator-activated receptors under epigenetic control in placental metabolism and fetal development. Am. J. Physiol. Endocrinol. Metab..

[68] Monsalve F.A., Pyarasani R.D., Delgado-Lopez F., Moore-Carrasco R. (2013). Peroxisome proliferator-activated receptor targets for the treatment of metabolic diseases. Mediat. Inflamm..

[69] Yessoufou A., Wahli W. (2010). Multifaceted roles of peroxisome proliferator-activated receptors (PPARs) at the cellular and whole organism levels. Swiss Med. Wkly..

[70] Emilsson V., Thorleifsson G., Zhang B., Leonardson A.S., Zink F., Zhu J., Carlson S., Helgason A., Walters G.B., Gunnarsdottir S. (2008). Genetics of gene expression and its effect on disease. Nature.

[71] Lin Q., Gao Z., Alarcon R.M., Ye J., Yun Z. (2009). A role of miR-27 in the regulation of adipogenesis. FEBS J..

[72] Takanabe R., Ono K., Abe Y., Takaya T., Horie T., Wada H., Kita T., Satoh N., Shimatsu A., Hasegawa K. (2008). Up-regulated expression of microRNA-143 in association with obesity in adipose tissue of mice fed high-fat diet. Biochem. Biophys. Res. Commun..

[73] Maeda A., Gohda T., Funabiki K., Horikoshi S., Tomino Y. (2004). Peroxisome proliferator-activated receptor γ gene polymorphism is associated with serum triglyceride levels and body mass index in Japanese type 2 diabetic patients. J. Clin. Lab. Anal..

[74] Garaulet M., Smith C.E., Hernández-González T., Lee Y.-C., Ordovás J.M. (2011). PPARγ Pro12Ala interacts with fat intake for obesity and weight loss in a behavioural treatment based on the Mediterranean diet. Mol. Nutr. Food Res..

[75] Rühl R., Landrier J.F. (2016). Dietary regulation of adiponectin by direct and indirect lipid activators of nuclear hormone receptors. Mol. Nutr. Food Res..

[76] Ding G., Cheng L., Qin Q., Frontin S., Yang Q. (2006). PPARδ modulates lipopolysaccharide-induced TNF-α inflammation signaling in cultured cardiomyocytes. J. Mol. Cell. Cardiol..

[77] Palomer X., Barroso E., Zarei M., Botteri G., Vázquez-Carrera M. (2016). PPARβ/δ and lipid metabolism in the heart. Biochim. Biophys. Acta.

[78] Andriankaja O.M., Galicia J., Dong G., Xiao W., Alawi F., Graves D.T. (2012). Gene expression dynamics during diabetic periodontitis. J. Dent. Res..

[79] Salvi G.E., Beck J.D., Offenbacher S. (1998). PGE2, IL-1β, and TNF-α responses in diabetics as modifiers of periodontal disease expression. Ann. Periodontol..

[80] Nareika A., Maldonado A., He L., Game B.A., Slate E.H., Sanders J.J., London S.D., Lopes-Virella M.F., Huang Y. (2007). High glucose-boosted inflammatory responses to lipopolysaccharide are suppressed by statin. J. Periodontal Res..

